# Identification of the Potential Key Long Non-coding RNAs in Aged Mice With Postoperative Cognitive Dysfunction

**DOI:** 10.3389/fnagi.2019.00181

**Published:** 2019-07-17

**Authors:** Ming Li, Chan Chen, Weiyi Zhang, Rui Gao, Qiao Wang, Hai Chen, Shu Zhang, Xiaobo Mao, Mathis Leblanc, Adam Behensky, Zheng Zhang, Lu Gan, Hai Yu, Tao Zhu, Jin Liu

**Affiliations:** ^1^Department of Anesthesiology and Translational Neuroscience Center, West China Hospital, Sichuan University, Chengdu, China; ^2^Department of Emergency Medicine, West China Hospital, Sichuan University, Chengdu, China; ^3^Institute of Cell Engineering, Department of Neurology, School of Medicine, Johns Hopkins University, Baltimore, MD, United States

**Keywords:** long non-coding RNA, microarray, postoperative cognitive dysfunction, aging, inflammation, apoptosis

## Abstract

Postoperative cognitive dysfunction (POCD) is a significant complication of surgery, particularly in elderly patients. Emerging researches showed that long non-coding RNA (lncRNA) may play a vital role in the pathogenesis of POCD. Here we aimed to identify potential key lncRNAs involved in the development of POCD. LncRNA and mRNA expression profiles in hippocampal tissues from POCD and control mice were analyzed by microarray assay. Gene ontology (GO) and KEGG pathway enrichment analyses were conducted to probe the functions of dysregulated genes. Then, important factors of the mainly affected biological processes were measured in the hippocampus. Correlated coding–non-coding co-expression (CNC) networks were constructed. Finally, the potential key pairs of lncRNA and target mRNA implicated in POCD were probed. Our data showed that 868 differentially expressed lncRNAs and 690 differentially expressed mRNAs were identified in total. GO and KEGG analyses indicated that the differentially expressed genes were mainly associated with inflammatory and apoptotic signaling pathways. Surgery-induced inflammatory cytokines and apoptosis were significantly increased in hippocampal tissues of aged mice. In CNC network analysis, we found that LncRNA uc009qbj.1 was positively correlated with apoptosis-associated gene *Vrk2* level. LncRNA ENSMUST00000174338 correlated positively with expression of the inflammation and apoptosis-associated gene *Smad7*. LncRNA NONMMUT00000123687 mediated gene expression by binding the inflammation-regulated transcription factor *Meis2*. Our results suggested that these potential key lncRNAs and mRNAs may play a crucial role in the development of POCD through mediating neuronal inflammation or apoptosis.

## Introduction

Postoperative cognitive dysfunction (POCD) is increasingly recognized as an important complication of major surgical procedures, especially in elderly patients (Moller et al., 1998; Newman et al., 2001). It is characterized by a persistent decline of cognitive performance after surgery, such as memory, information processing, and executive dysfunction (Monk and Price, 2011). Unfortunately, to date, the specific mechanisms underlying POCD remain unclear. These mainly include neuroinflammation (Skvarc et al., 2018), amyloid–β (Aβ) deposition (Xu et al., 2014), hyperphosphorylation of tau proteins (Tan et al., 2010), and neuronal apoptosis (Yon et al., 2005). Adults aged 65 or older are expected to be the largest surgical population by 2020. The number of patients at risk of developing POCD is bound to increase dramatically, which leads to increased surgical morbidity and mortality (Steinmetz et al., 2009). Patients with POCD often have decreased quality of life, prolonged hospital stay and increased cost of hospitalization (Moller et al., 1998; Steinmetz et al., 2009). Therefore, it is urgently needed to explore the molecular mechanisms of POCD for developing potential therapeutic targets for prevention, diagnosis and treatment.

As an important member of the non-coding RNAs, long non-coding RNA (lncRNAs) are currently defined as transcripts of more than 200 nucleotides without evident protein-coding function. According to their transcriptional location the genome, lncRNA can be further classified into three subcategories: (A) long intergenic non-coding RNAs (lincRNAs) transcribed from intergenic regions to regulate the expressions of adjacent genes, (B) lncRNAs transcribed from the gene regulatory regions, and (C) the rest of lncRNAs transcribed at some specific chromosomal regions to modulate epigenetic modifications of DNA (Chen and Zhou, 2017). Previous studies have shown that lncRNA-related dysfunction played critical roles in various diseases including cancers (Spizzo et al., 2012), cardiovascular diseases (Schonrock et al., 2012), and neurodegeneration diseases (Johnson, 2012). Notably, in 2008, *BACE1*-AS, a lncRNA, was found to be directly related to the increase of Aβ 1-42 in Alzheimer’s disease (AD) (Faghihi et al., 2008). Moreover, recent studies indicated that lncRNAs could serve as negative feedback regulators of excessive inflammation and engage in the progress of neuron apoptosis (Du et al., 2017; Gu et al., 2018). These biological processes are pathologically relevant to the development of POCD. Therefore, we hypothesize that lncRNAs may also have important functions in the pathogenesis of POCD.

In the present study, to identify novel targets for further study of POCD, we profiled differentially expressed lncRNAs and mRNAs from hippocampal tissue samples between POCD and paired control mice. Then, functional enrichment analyses were applied to investigate the principal functions of differentially expressed genes, and novel co-expression networks of non-coding–coding genes were constructed for predictions on the function of lncRNAs. Finally, we identified some potential key pairs of lncRNAs and target mRNAs, which may exert a vital role in the development of POCD. Our findings may help to provide some insights for future research in POCD.

## Materials and Methods

### Animal Experiments

We randomly grouped male C57BL/6 mice (SIPPR/BK Lab Animal Ltd., Nanjing, China) (weight, 30–35 g; age, 16–18 months) and assigned them to a specific experiment. Mice were housed in polypropylene cages under standardized conditions with free access to food and water. Data from mice determined to be unhealthy were ruled out from analysis. All procedures were in accordance with the National Institute of Health Guidelines for the Care and Use of Laboratory Animals and approved by West China Hospital Institutional Animal Care and Use Committee. Investigators who treated animals learned about the treatment groups and collected samples, which were then analyzed by other investigators who were blinded to the specific treatment.

### Animal Model of POCD

The surgical procedure of unilateral nephrectomy was performed as previously described (Vizcaychipi et al., 2014). Briefly, the experimental mice were anesthetized with an intraperitoneal injection of ketamine and xylazine mixture (120 and 4 mg/kg, respectively). A midline longitudinal incision was made and the left kidney was carefully resected. Finally, 0.2% subcutaneous ropivacaine was injected before the incision was closed. After surgery, mice were given 0.5 mL of saline intraperitoneally. Body temperature during the surgery was maintained at (37.0 ± 0.5)°C using a heating pad. Animals recovered in an incubator maintained at 37°C, and then was moved back to their home cages. The control group received no specific treatment.

### Behavioral Tests

#### Fear Conditioning Test

The FCT studies were performed according to a previous study with minor modifications (Xu et al., 2014). The pairing in the FCT (Ugo Basile, Varese, Italy) was performed on the first day post-surgery to simulate the situation that patients may be difficult to learn new things after surgery. Each mouse was able to probe the FCT chamber for 100 s before the onset of a 2 Hz pulsed sound (80 dB, 3,600 Hz) for 20 s. Then, a slight foot shock (0.8 mA for 2 s) was immediately presented after the end of the tone. We repeated the procedure once and moved out the mouse 30 s later. In total, it was 274 s of training time. The first context test of FCT was performed 30 min after the end of training. The experiment mice were allowed to stay in the same chamber for another 274 s without cue tone and foot shock exploration. Then, we conducted the first tone test 90 min after training. Each mouse was placed in a context different chamber for 360 s. The same tone was given for the second 180 s without electrical stimulation. Cognitive function in the context test and tone test was evaluated by calculating the percentage of freezing time. The same cohorts of experimental mice were tested repeatedly in the FCT on day 1 and day 3 after operation.

#### Open Field Test

To assess the locomotor activity and anxiety of mice, OFT was performed by exploring a new environment (Terrando et al., 2016). Briefly, mice were directly placed into the middle of the open field (60 cm × 40 cm × 20 cm, length × width × height). The total area was divided into 2 squares. The central area accounted for 60% of the total area as previously defined (Balazsfi et al., 2018). Movements were recorded for 5 min using a digital camera. Measures of activity (average velocity, center square duration, and distance traveled) were recorded and analyzed using the CageCenter tracking software. The number of rearings was counted manually by a researcher.

### RNA Extraction

The hippocampal tissues of the surgery and control group mice were harvested on day 3 after the surgery and stored at – 80°C until use. Total RNA was extracted from the two groups according to the manufacturer’s instructions using Trizol reagent (Invitrogen, Carlsbad, CA, United States). The RNA integrity of each sample was estimated using standard denaturing agarose gel electrophoresis and quantified via a NanoDrop spectrophotometer (NanoDrop, Wilmington, DE, United States).

### Quantitative Real-Time PCR

Total RNA was extracted from hippocampus of these experimental mice using TRIzol^®^ Reagent (Invitrogen Life Technologies), and then a QuantiTect Reverse Transcription Kit (Bio-Rad, United States) was used for reverse transcription of the RNA and cDNA synthesis according to the manufacturer’s instructions. Quantitative real-time PCR (qRT-PCR) was conducted by an Eppendorf RT-PCR system (Hauppauge, NY). 18s mRNA was utilized for the control gene to normalize the data of each sample. The specific primer pairs in the study were listed in Supplementary Table S1. Relative gene expression was determined by employing the 2^–ΔΔCt^ method.

### TUNEL Staining

Neuronal apoptosis was measured utilizing terminal deoxynucleotidyl transferase dUTP nick end labeling (TUNEL) assay. Briefly, Mouse was anesthetized and thoracic cavity was opened to expose the heart. Then, 0.9% 20 ml normal saline was transcardially perfused, followed by 4% 20 ml paraformaldehyde infusions. The brains were harvested and post-fixed using 4% paraformaldehyde for 24 h, followed by dehydrated and dissected at a thickness of 10-μm. To determine the number of apoptotic cells, an In Situ Cell Death Detection Kit (TMR Green; Roche Diagnostics, Germany) and DAPI staining were utilized in accordance with the manufacturer’s protocol. TUNEL-positive nuclei were identified through the co-localization of presenting both the TUNEL signal and DAPI. The positive nuclei from 5 microscopic fields in each section of hippocampal CA1 region (400×) were counted, and the percentage of which was calculated.

### Microarray

DNA microarray: For the global analysis of mouse mRNAs and lncRNAs, the Arraystar mouse lncRNA microarray V3.0 was designed. By querying authoritative data sources, such as Ensembl, UCSC Knowngenes, RefSeq and so on, about 35,923 lncRNAs were obtained. In every microarray research, total RNA of 3 mouse hippocampal samples from the surgery and control groups were pooled, respectively, and then used for hybridization. This microarray assay was repeated twice in the 2 different groups of mouse-derived hippocampal samples.

RNA labeling and array hybridization: Sample labeling and array hybridization were conducted in the light of the Agilent gene expression analysis protocol (Agilent Technology) with some minor modifications. Briefly, mRNA was purified from total RNA after removing rRNA, and then amplified and transcribed into fluorescent cRNA along the whole length of the transcripts without 3’ bias using a random priming method. The labeled cRNAs were purified through RNeasy Mini Kit (Qiagen). The concentration and specific activity of the labeled cRNAs (pmol Cy3/μg cRNA) were measured by NanoDrop ND-1000. 1 μg of each labeled cRNA was fragmented by first adding 5 μl 10 × Blocking Agent and 1 μl of 25 × Fragmentation Buffer, then heating the mixture at 60°C for 30 min, and finally the labeled cRNA was dilute by adding 25 μl 2 × GE Hybridization Buffer. 50 μl of hybridization solution was then allocated to the gasket slide which was set up with lncRNA microarray slide. Subsequently, the slide was placed into an Agilent hybridization oven and incubated for 17 h at 65°C. Then, the microarray was washed, fixed and scanned by Agilent microarray scanner (Agilent p/n G2565BA).

### Data Analysis

The collected array images were analyzed by Agilent feature extraction software (version 11.0.1.1). For quantile normalization and subsequent data processing, the GeneSpring GX v12.1 software package (Agilent Technologies) was utilized. In further data analysis, LncRNAs and mRNAs that 2 out of two samples had flags in Present or Marginal (“All Targets Value”) were chosen after quantile normalization of the raw data. Fold change filtering was employed for differentially expressed lncRNAs and mRNAs identification between the two groups. Gene ontology (GO) and KEGG pathway enrichment analysis were conducted through the standard computation method. Gene Ontologies are organized into a hierarchy structure of annotation terms in order to promote an analysis and interpretation at different levels. The top-level ontologies are biological process, cellular component, and molecular function (Beissbarth, 2006). Thus, GO database^1^ analysis was employed to reflect genetic regulatory systems based on the differentially expressed mRNAs in the biological process, cellular component and molecular function classification. Fisher’s exact test was utilized for determining if the similarity between differential expression and GO annotation list was greater than assessed by chance. KEGG database^2^ analysis was applied to identify the potential key pathways related to the differentially expressed genes. Fisher’s exact test was also used for the enrichment Fisher-*P*-value or Hypergeometric-*P*-value of KEGG pathway. *P* < 0.05 indicated that GO terms and KEGG pathways of differentially expressed genes were significantly enriched. *P*-value was corrected using Benjamini–Hochberg false discovery rate (FDR). The microarray analysis was performed by KangChen Bio-tech (Shanghai, China).

### Co-expression Network Construction

The co-expression networks of lncRNAs and related mRNA were constructed according to the correlation between the differentially expressed lncRNAs and mRNAs. Pearson correlation coefficients (PCC) > 0.95 or < –0.95 between lncRNAs and mRNAs were picked out to draw networks by program Cytoscape (Dong et al., 2014).

### Western Blot Analysis

Protein was extracted as previously described (Zhang et al., 2012). Equal amounts of protein (100 μg) was resolved by 10% sodium dodecyl sulfate-polyacrylamide gel electrophoresis (SDS-PAGE), followed by transferred onto polyvinylidenedifluoride (PVDF) membrane (Millipore, Billerica, MA, United States). The membrane was blocked in 5% skim milk, and then incubated at 4°C overnight with primary antibodies against cleaved caspase-3 (1:100; Proteintech, Wuhan, China) and α-tubulin (1:100; Proteintech, Wuhan, China), followed by incubation with HRP-labeled secondary antibodies (1:5000; Zsbio, Beijing, China). ECL fluorescent detection reagent (Millipore, Billerica, MA, United States) was used for immunoreactivity and the density of immunoreactive strips was analyzed through NIH ImageJ software.

### Statistical Analysis

Statistical analyses were conducted with Prism 7.0 for Windows (GraphPad, La Jolla, CA). All data are expressed as means ± SEM. For microarray data, the statistical significance in fold change was analyzed utilizing the Student’s *t* test and FDR was calculated to correct the *P*-value. Differentially expressed lncRNAs and mRNAs were screened using fold changes greater than or equal to 2 as thresholds. For other data [Western Blot (WB), qRT-PCR], statistical significance between the two groups was determined by Unpaired 2-tailed Student’s *t* test. A value of *P* < 0.05 was considered statistically significant.

## Results

### Unilateral Nephrectomy Induced Anxiety-Like Behavior and Cognitive Deficits in Aged Mice

**FIGURE 1:**
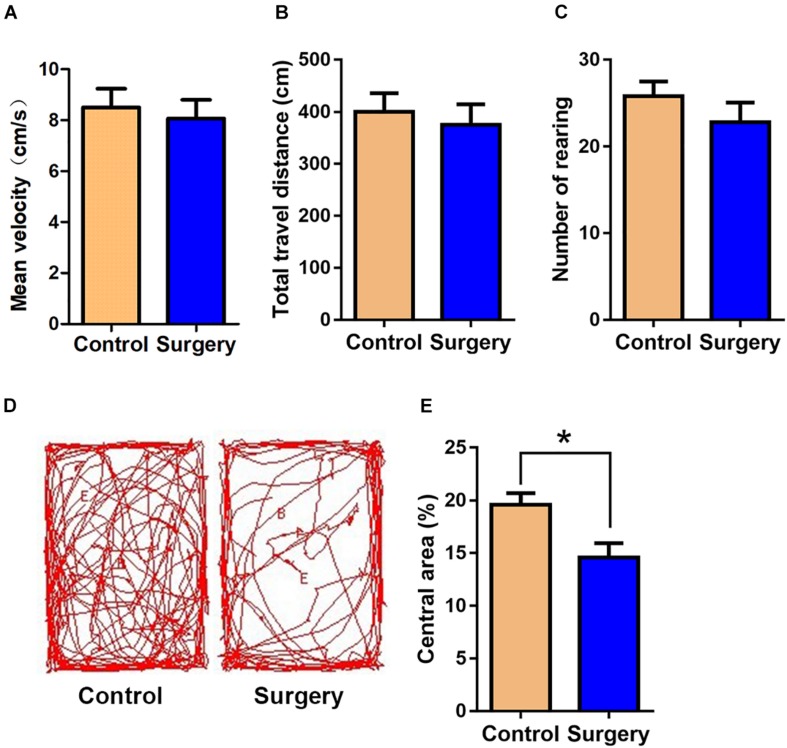
Surgical trauma induces anxiety-like behavior in aged mice. The time in the central area was taken as measures of anxiety and exploratory behavior in the open-field test. **(A)** Mean velocity. **(B)** Total travel distance. **(C)** Rearing activity. **(D)** Entries into the center. **(E)** % central area. Data are presented as means ± SEM; ^*^P < 0.05, vs. Control, Student’s *t*-test, *N* = 10 for all analyses.

**FIGURE 2:**
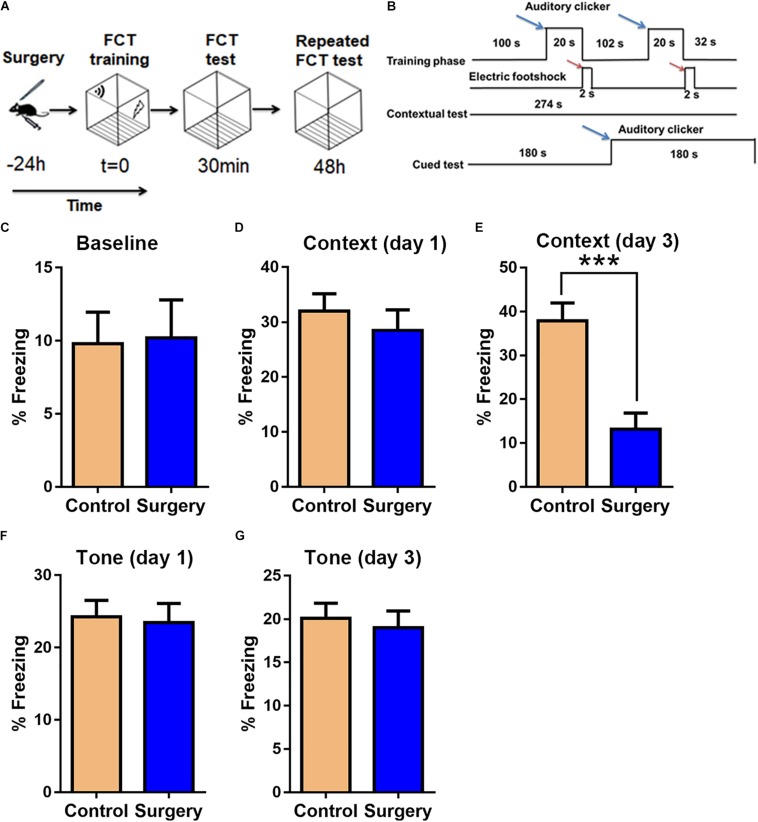
Unilateral nephrectomy impairs associative cognitive function on day 3 post-surgery. **(A)** Fear conditioning test (FCT) design. **(B)** FCT workflow. Blue arrows indicate sound stimuli and red arrows indicate electric shock stimuli. **(C)** Baseline freezing levels. **(D,E)** Freezing levels in the contextual test on day 1 and day 3 post-surgery. **(F,G)** Freezing levels in the cued tone test on day 1 and day 3 post-surgery. Data are presented as means ± SEM; ^∗∗∗^ *P* < 0.001, vs. Control, Student’s *t*-test, *N* = 12 for all analyses.

There was little difference between control and surgery mice in the case of mean velocity, total travel distance, or rearing activity in the open field test (OFT) at day 1 post-surgery (*P* > 0.05, Figures 1A–C). However, the center duration of the surgery mice was significantly shorter compared with that of controls at day 1 following surgery (*P* < 0.05, Figures 1D,E). The fear conditioning test (FCT) design and workflow are described in Figures 2A,B. In the contextual test, the average freezing time was not different between the surgery and control group mice on day 1 after nephrectomy (*P* > 0.05, Figure 2D). However, on day 3 after the operation, contextual memory in the surgery group mice was significantly decreased compared with the control group mice, as evidenced by a shorter average freezing time (*P* < 0.001, Figure 2E), while baseline and cued freezing levels were little difference between the two groups (*P* > 0.05, Figures 2C,F,G). These data demonstrated that unilateral nephrectomy induced significant behavioral deficits in aged mice on the third day post-surgery.

### Overview of lncRNA and mRNA Expression Profiles

**FIGURE 3:**
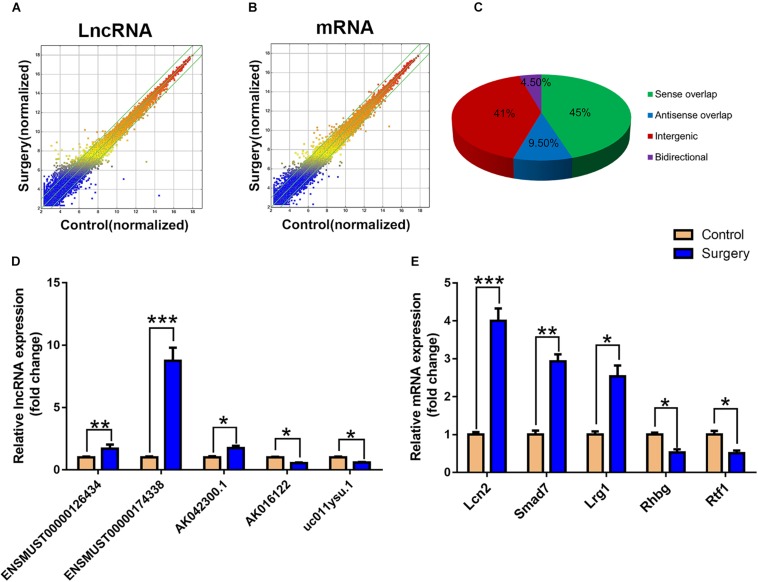
LncRNA and mRNA expression profiles. **(A)** The scatterplots of lncRNA expression profiles. **(B)** The scatterplots of mRNA expression profiles. The scatterplots of lncRNA and mRNA were used to assess the lncRNA and mRNA expression variations between the surgery group and the control group. The green lines represent the fold change (the default value is 2 times). **(C)** Subgroup analysis of differentially expressed lncRNAs was based on their gene mapping and the relationships between adjacent protein-encoding genes. **(D)** Relative expression of five lncRNAs. **(E)** Relative expression of five mRNAs. Data are presented as means ± SEM; ^*^P < 0.05, ^∗∗^*P* < 0.01, ^∗∗∗^*P* < 0.001, vs. Control, Student’s *t*-test, *N* = 10 for all analyses.

The scatter plots were a visualization method used for assessing the lncRNA and mRNA expression variations between surgery and control samples (Figures 3A,B). Compared with the lncRNAs in the control group, 868 lncRNAs were differentially expressed in the surgery group; 496 were upregulated, and 372 were downregulated. ENSMUST00000121407 was the most up-regulated lncRNA, and NR_037988 was the most down-regulated lncRNA (Table 1). Depending on the genomic location that implies that lncRNAs may have the potential functions in regulating their adjacent protein-coding genes, each of the differentially expressed lncRNAs was classified into 1 of 4 categories (sense overlap, antisense overlap, intergenic, bidirectional) (Figure 3C). About 40% of these lncRNAs belong to the intergenic lncRNA category, of which many were functional and conserved in mammals (Guttman et al., 2009).

**TABLE 1:** Top 10 differentially expressed lncRNAs.

**Top 10 differentially expressed lncRNAs (Surgery vs. Control)**			
**Up-regulated lncRNAs**		**Down-regulated lncRNAs**	
**LncRNA seqname**	**Fold change**	**LncRNA seqname**	**Fold change**
ENSMUST00000121407	44.0738891	NR_037988	75.646121
AK134221	38.3875292	uc009rwl.1	49.2329881
TCONS_00022552	23.5318102	ENSMUST00000077582	19.9324922
AK144133	15.5019307	ENSMUST00000131864	16.4125705
ENSMUST00000174338	15.1467424	AK155619	9.4494824
TCONS_00010795	14.521925	uc011ysu.1	8.4173669
AK030988	12.0249851	ENSMUST00000181342	7.6085261
mouselincRNA0065+	11.7505852	ENSMUST00000156958	6.6236011
humanlincRNA0860+	10.7063974	AK016122	6.5157073
AK149831	9.6826759	AK036292	6.1184775

Additionally, the mRNA profiles between the two groups were also analyzed. In comparison with the mRNAs in the control mice, 690 mRNAs were differentially expressed in surgery group; 249 were upregulated and 441 were downregulated. Cxcl2 was the most up-regulated mRNA, and Tmem591 was the most downregulated mRNA (Table 2). To further evaluate the consistency of the microarray, 5 pairs of differentially expressed lncRNAs and mRNAs were randomly selected and analyzed further with qRT-qPCR in 10 pairs of mice. LncRNAs ENSMUST00000126434, ENSMUST00000174338 and AK042300.1 were upregulated, while AK016122 and uc011ysu.1 were downregulated (Figure 3D). *Lrg1, Smad7* and *Lcn2* mRNAs were upregulated, while *Rhbg* and *Rtf1* were downregulated (Figure 3E). qRT-PCR analysis results were in accordance with our microarray analyses, thus indicating the reliability of the microarray data. The lncRNA and mRNA raw data have been uploaded into Gene Expression Omnibus (GEO), and the GEO accession number is GSE113738. The full information of all the significant dysregulated lncRNAs and mRNAs of microarray analysis has been provided in Supplementary Table S2.

**TABLE 2:** Top 10 differentially expressed mRNAs.

**Top 10 differentially expressed mRNAs (Surgery VS Control)**			
**Up-regulated mRNAs**		**Down-regulated mRNAs**	
**mRNA seqname**	**Fold change**	**mRNA seqname**	**Fold change**
Cxcl2	10.4449494	Tmem59l	19.2085272
Slc47a1	10.0731291	Prr23a	18.1825571
Cdh1	8.6121235	Pdzk1ip1	16.1521174
AU018091	8.3063289	S100a8	8.2135619
Lcn2	8.1802254	Vmn1r219	7.943022
Dapl1	8.0599048	Celsr1	7.6575533
Areg	6.5823576	Pask	7.2041494
Emilin3	6.5731322	Rcc2	6.5116538
H2-M10.3	6.3862432	Pafah1b1	5.9276695
Tfap2a	6.1562862	Pop1	5.6833151

### GO and KEGG Pathway Analysis

Functional enrichment analysis of these differentially expressed mRNAs was conducted. In GO biological process analysis, the downregulated and upregulated functional GO terms were mainly linked to the immune/inflammatory system and apoptotic process. Additionally, as shown in Table 3, the top 10 upregulated biological processes were mainly involved in the chemotaxis/migration of inflammatory cells (T-cell, lymphocyte, and monocyte), and the top 6 downregulated biological processes included mainly synaptic transmission, amyloid precursor protein (APP) catabolic process and interleukin-10 (IL-10) production. For GO molecular function analysis, the most significant enriched of the upregulated and downregulated functional GO terms were carbohydrate phosphatase activity (GO:0019203) and Ran GTPase binding (GO:0034595), respectively (Supplementary Figures S1A,B). With regards to GO cellular component analysis, the most significant enriched of the upregulated and downregulated functional GO terms were basolateral plasma membrane (GO:0016323) and zymogen granule (GO:0042588), respectively (Supplementary Figures S1C,D). The full information of all the significant enrichment GO categories was shown in Supplementary Table S3.

**TABLE 3:** Top 10 GO biological processes of upregulated target genes and top 6 biological processes of downregulated target genes (surgery vs. control).

**GO.ID**	**GO Term**	**Fold enrichment**	***P* value**
**Upregulated target genes**			
GO:0090025	regulation of monocyte chemotaxis	> 12	< 0.01
GO:0033631	cell-cell adhesion mediated by integrin	> 12	< 0.01
GO:0010820	positive regulation of T cell chemotaxis	> 12	< 0.01
GO:0010819	regulation of T cell chemotaxis	> 12	< 0.01
GO:0036230	granulocyte activation	> 12	< 0.01
GO:0002548	monocyte chemotaxis	> 12	< 0.01
GO:2000403	positive regulation of lymphocyte migration	> 12	< 0.01
GO:1902337	regulation of apoptotic process	> 12	< 0.01
GO:0006925	inflammatory cell apoptotic process	> 12	0.011
GO:1901623	regulation of lymphocyte chemotaxis	> 12	0.01
**Downregulated target genes**			
GO:0050805	negative regulation of synaptic transmission	> 5	0.001
GO:0032607	interferon-alpha production	> 5	0.001
GO:0060292	long term synaptic depression	> 5	0.029
GO:0042987	amyloid precursor protein catabolic process	> 5	0.03
GO:1902803	regulation of synaptic vesicle transport	> 5	0.036
GO:0032733	regulation of interleukin-10 production	> 5	0.044

**FIGURE 4:**
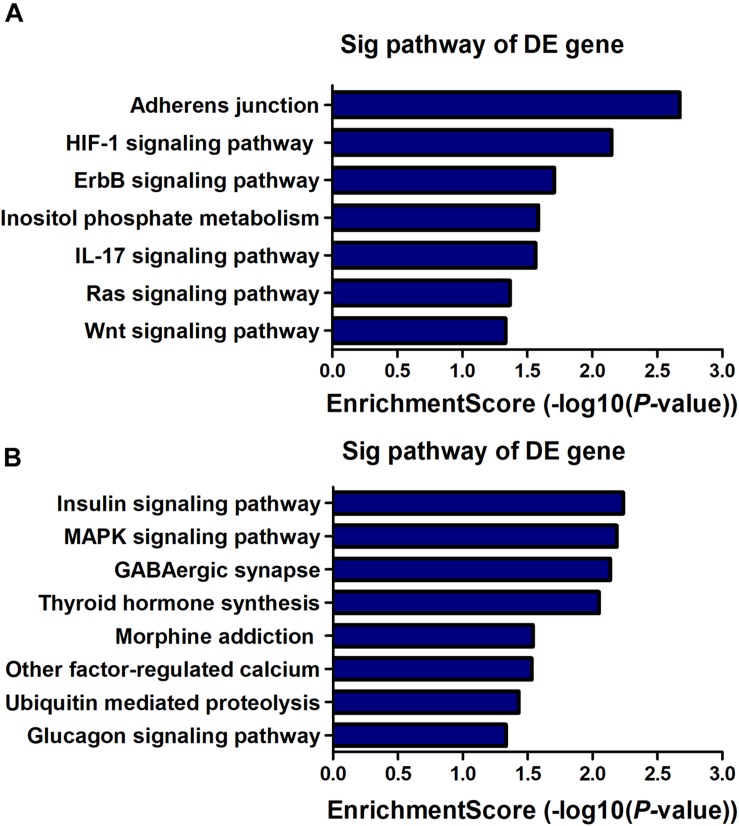
KEGG pathway enrichment analysis is a functional analysis for mRNAs. The *P*-value (EASE-score, Fisher-*P-*value or Hypergeometric-*P*-value) indicates the significance of the pathway correlated to the conditions. The lower the *P*-value, the more significant the pathway is (The recommended *P*-value cut-off is 0.05). **(A)** Pathways involved in up-regulated transcripts. **(B)** Pathways involved in down-regulated transcripts.

KEGG pathway analysis revealed that 7 pathways corresponded to up-regulated transcripts (Figure 4A) and that the most enriched network was “Adherens junction (mouse)” (Fisher *P*-value = 2.12E-03) composed of 8 targeted genes. Moreover, the pathway analysis also indicated that 8 pathways corresponded to downregulated transcripts (Figure 4B) and that the most enriched network was “Insulin signaling pathway (mouse)” (Fisher *P*-value = 5.80E-03) composed of 11 targeted genes. Among these pathways, the gene category “IL-17 signaling pathway” has been reported to play a pivotal role in cognitive impairment (Tian et al., 2015); the gene category “ErbB signaling pathway” has been found to be implicated in inhibition of long-term potentiation (LTP) in hippocampus (Pitcher et al., 2008). Additionally, the gene category “Hypoxia-inducible factor-1 (HIF-1) signaling pathway” has been shown to be associated with the pathogenesis of neurodegenerative diseases, such as AD, Huntington’s disease (HD) and Parkinson’s disease (PD) (Zhang et al., 2011). The full information of all the significant enrichment KEGG pathways was shown in Supplementary Table S4.

### Surgery-Induced Inflammatory Cytokines and Neuronal Apoptosis Were Increased in Hippocampal Tissue of Aged Mice

**FIGURE 5:**
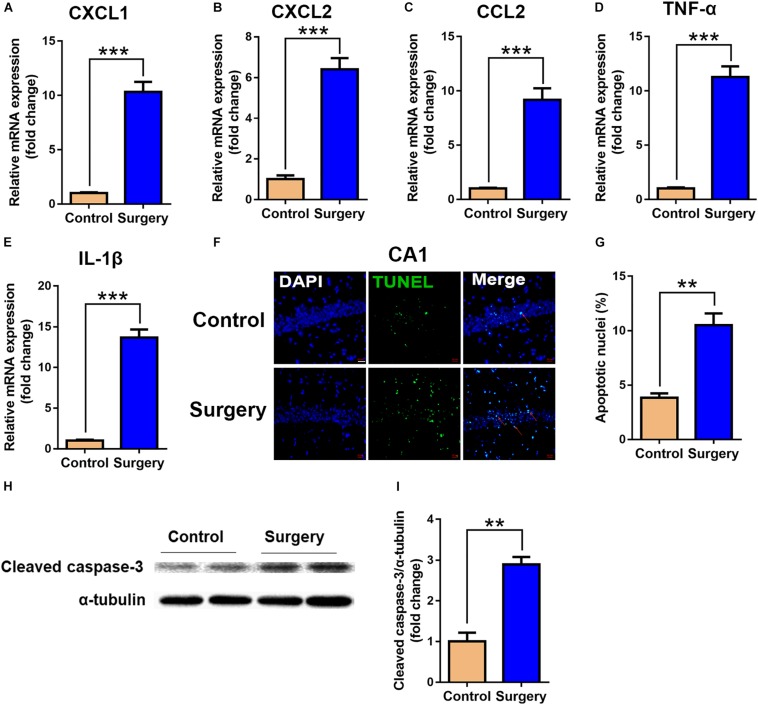
Expression of inflammatory cytokines and neuronal apoptosis were significantly enhanced in hippocampal tissue after surgery. Hippocampal tissues were harvested on day 3 after surgery from the two groups. **(A–E)** mRNA expression of CXCL1 **(A),** CXCL2 **(B)**, CCL2 **(C)**, TNF-α **(D)** and IL-1β **(E)**. Proinflammatory cytokine expression was measured by qRT-PCR. **(F)** Representative images of TUNEL assay in the CA1 of the hippocampus. Scale bar, 20 μm. **(G)** Quantitative analysis of TUNEL-positive cells. Red arrows point to representative TUNEL-positive cells. The cells were counterstained with 4’, 6-diamidino-2-phenylindole (DAPI). **(H)** Protein levels of cleaved caspase-3. **(I)** Quantification of protein levels of cleaved caspase-3. Data are presented as means ± SEM; ^∗∗^*P* < 0.01, ^∗∗∗^ *P* < 0.001, vs. Control, Student’s *t*-test, *N* = 6 for all analyses.

To validate the functional enrichment analyses results that the differentially expressed genes were mainly related to inflammatory and apoptotic signaling pathways, we measured the significant factors implicated in the two GO terms in the hippocampus. The results showed that unilateral nephrectomy significantly increased the expression of inflammatory chemokine (C-X-C motif) ligand 1 (CXCL1) and ligand 2 (CXCL2), and chemokine (C-C motif) ligand 2 (CCL2) at mRNA levels in hippocampus compared to that of the control group (*P* < 0.001, Figures 5A–C). Also, the expression of inflammatory cytokines in the surgery group, including tumor necrosis factor α (TNF-α) and interleukin-1β (IL-1β), was dramatically enhanced compared with the control group (*P* < 0.001, Figures 5D,E). Moreover, compared with the control group, the ratio of apoptotic neurons in the hippocampal tissue was significantly increased in the surgery group via TUNEL staining (*P* < 0.001, Figures 5F,G). Consistently, the level of cleaved caspase-3 proteins, which is a key mediator of cellular apoptosis, was also significantly increased in the surgery group through WB analysis (*P* < 0.01, Figures 5H,I). Our results showed that inflammatory response and neuronal apoptosis were markedly enhanced on day 3 after surgery in the hippocampus of the aged mice.

### LncRNA/mRNA Co-expression and Function Prediction

**FIGURE 6:**
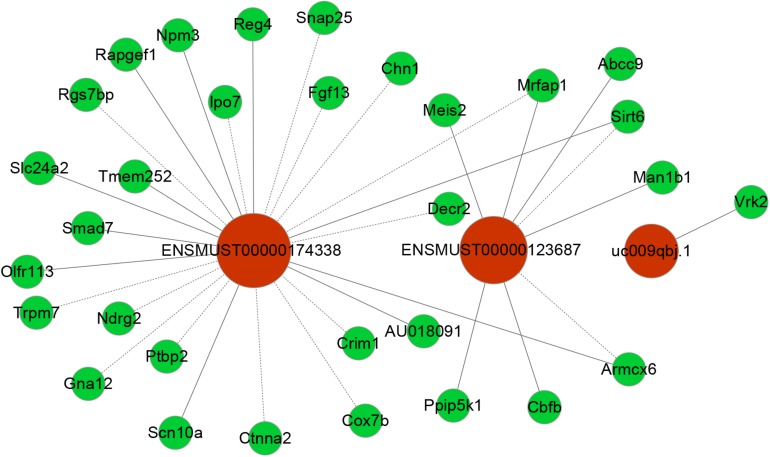
Co-expression networks of third lncRNAs with associated mRNAs. Co-expressed lncRNA-mRNA pairs were identified through strict screening criteria (correlation coefficients > 0.95 or < –0.95, *P* < 0.01). The differentially expressed lncRNAs were predicted to function by regulating the closely related mRNA. Red circles represent lncRNAs, and green circles denote mRNAs. Solid and dotted lines represent positive correlation and negative correlation, respectively.

To date, predictions on the function of lncRNAs were mainly dependent on their co-expression with corresponding coding genes. Thus, the co-expression networks of differentially expressed lncRNA-mRNA were constructed based on the correlation analysis. Through a rigorous screening process, co-expressed lncRNA-mRNA gene pairs (PCC > 0.95 or < –0.95, and *P* < 0.01) were selected to draw the networks (Figure 6). LncRNA ENSMUST00000174338 was positively correlated with *Smad7*, *Sirt6*, *Reg4*, *Npm3*, and *Rapgef1* levels, and negatively correlated with *Ndrg2*, *Chn1*, *Lpo7*, and *Crim1* expression. LncRNA uc009qbj.1 was positively correlated with *Vrk2* level and lncRNA NONMMUT00000123687 was positively correlated with *Abcc9*, *Meis2*, *Cbfb*, and *Man1b1* levels, and negatively correlated with *Armcx6* and *Sirt6* expression. These networks showed that one lncRNA could correlate with one to tens of mRNAs. Co-expressed mRNAs were mainly implicated in inflammation and neuronal apoptosis pathways.

### Predicting the Potential Key lncRNA-mRNA Pairs Involved in POCD

At present, the function of lncRNA is mainly achieved by acting on target genes in the form of cis or trans (Wei et al., 2017). Combining lncRNA and mRNA co-expression networks, potential targets of lncRNAs was predicted according to cis or trans prediction. The cis-prediction screened the pairs of lncRNA-mRNA based on the distance within 10 kb of one another, while trans-prediction identified the pairs of lncRNA-mRNA using the method of calculating combined energy (if the combined energy value < 30, it is determined to trans). The results indicated that lncRNA ENSMUST00000174338 regulated the inflammation and apoptosis-associated gene *Smad7* in cis. LncRNA NONMMUT00000123687 mediated gene expression by binding the inflammation-regulated transcription factor *Meis2* in trans.

## Discussion

Emerging researches have shown that lncRNAs may be involved in the pathogenesis of POCD, but the underlying mechanisms remain unclear (Wei et al., 2017; Zhang et al., 2018). This study was an exploratory analysis on which lncRNAs were involved in the pathogenesis of POCD. We established a POCD mouse model based on previous research (Vizcaychipi et al., 2014), which has been well established and confirmed in our previous study (Chen et al., 2015). And we have identified the significantly dysregulated lncRNA and mRNA expression profiles in POCD compared with the control mice. In total, 868 differentially expressed lncRNAs and 690 expressed mRNAs were identified. GO and KEGG pathway enrichment analyses of the differentially expressed genes indicated that they were mainly linked to the progress of cellular inflammation and apoptosis. Furthermore, in the *in vivo* study, we demonstrated that surgery-induced inflammatory cytokines and apoptosis were increased in the hippocampus of aged mice. Importantly, we identify some potential key pairs of lncRNAs and target mRNAs, which may play a pivotal part in the pathogenesis of POCD through mediating neuronal inflammation or apoptosis.

Previously, unilateral nephrectomy has been reported for the establishment of POCD mouse model (Vizcaychipi et al., 2014; Chen et al., 2015). Again, in this study, we confirmed that this procedure modality could induce associative cognitive deficits on day 3 after surgery (Figure 2). And we applied microarray analyses to identify lncRNA and mRNA expression profiles in hippocampal tissues from POCD and control mice. Although similar research was performed on POCD in aged mice (Wei et al., 2017), there are some differences from our study. First, we utilized classical unilateral nephrectomy to establish the model of POCD instead of tibial fracture. Second, in this model of POCD, we further confirmed that surgery-induced neuroinflammation and apoptosis were significantly increased in aged mice. Third, in the study of Wei et al. (2017), they were mainly concerned with the comprehensive role of lncRNAs in POCD, while we focused on identification of the potential key pairs of lncRNAs and target mRNAs. Thus, our research further probed the possible roles of lncRNAs in the development of POCD.

LncRNA, as a regulatory factor, has recently been demonstrated to be closely related to excessive inflammation and neuronal apoptosis (Du et al., 2017; Gu et al., 2018). In addition, numerous studies indicated that neuroinflammation and neuronal apoptosis play an important role in POCD formation and development (Yon et al., 2005; Cibelli et al., 2010; Terrando et al., 2010). In the context of POCD, we found that many biological immune/inflammatory and apoptotic processes may be potentially targeted by the lncRNAs (Table 3). Regarding these biological processes, the regulation of T-cell/lymphocyte chemotaxis/migration was most affected. Activated immune cells could release a large number of pro-inflammatory cytokines, such as IL-6, IL-1β and TNF-α, participating in the early event in POCD formation (Terrando et al., 2010). KEGG pathway analysis for the differentially expressed genes also revealed some important pathways that could be involved in POCD, including IL-17 signaling pathway (Tian et al., 2015), ErbB signaling pathway (Pitcher et al., 2008), HIF-1 signaling pathway (Zhang et al., 2011), and Wnt signaling pathway (Zheng et al., 2016). These pathways were well known to be associated with occurrence and development of neurodegenerative diseases. We further confirmed that inflammatory cytokines and apoptosis relative indexes were markedly increased in this model of POCD compared with the control mice *in vivo*, which was consistent with previous findings (Zhang et al., 2016; Qi et al., 2017). It therefore could be hypothesized that lncRNAs deregulation may promote POCD formation and development through mediating neuronal inflammation and apoptosis.

However, it remains elusive which lncRNAs play a crucial role in the pathogenesis of POCD. Up to now, the functions for most lncRNAs are unclear. We usually predict the lncRNA function relying on its closely related mRNA functions in CNC network. *Smad7* gene is a member of the *Smad* family. Previous reports showed that Smad7 upregulation mediated the increase of TGF- β1 induced intestinal mucositis factors expression (Monteleone et al., 2001). In addition, it was found that the increase of Smad7 expression in the cerebral cortex mediated TGF- β1 induced neuronal apoptosis in the mouse model of AD (Salins et al., 2008). Notably, Smad7 downregulation could attenuate cellular inflammation and apoptosis (Hong et al., 2007; Salins et al., 2008; Monteleone et al., 2015). VRK2 protein, a member of the VRK (vaccinia-related kinase) family of protein kinases, can modulate apoptosis in two different ways by regulation of BAX gene expression and by its interaction with Bcl-xL (Monsalve et al., 2013). Meis2, a member of Meis family, is a homeodomain transcription factor. Recent study showed that Meis2 participated in intrinsic inflammatory signaling circuit (Jeong et al., 2017). More importantly, Meis2 knockdown in prefrontal cortex was associated with working memory defects (Jakovcevski et al., 2015). In the present study, we identified that the expression of Smad7 and VRK2 was remarkably upregulated and Meis2 was significantly downregulated accompanied by obvious neuroinflammatory response and cellular apoptosis in the hippocampus after unilateral nephrectomy. In our coding–non-coding co-expression (CNC) networks analysis, we found that lncRNA ENSMUST00000174338 correlated positively with expression of Smad7 and could regulate its expression in cis; LncRNA uc009qbj.1 was positively correlated with *Vrk2* level; LncRNA NONMMUT00000123687 correlated positively with expression of *Meis2*. Collectively, our findings suggested that these key lncRNAs may play a vital role in the formation and development of POCD through interacting with their corresponding coding genes.

Our study has some potential limitations. First, we only identified differentially expressed genes in aged mice with POCD on day 3 after surgery, when we have observed learning and memory dysfunction. Admittedly, we could not tell if gene expression would be changed at a longer time point. Second, the possible roles of lncRNA-mRNA axis in the development of POCD are mainly based on bioinformatics prediction, thus whether the identified differentially genes are the real initiators of POCD requires further experimental validation. Third, we only analyzed lncRNAs related to the development of POCD in nephrectomy, but did not verify if other types of surgery had similar effects.

In conclusion, through combining analysis of the Arraystar Mouse lncRNA/mRNA microarray, we have screened differentially expressed lncRNAs and mRNAs in hippocampus from POCD and control mice. Then, functional enrichment analyses were employed to evaluate the functions of these differentially expressed genes and their correlated pathways. Coding–non-coding gene expression networks were constructed to analyze non-coding gene functions. Finally, combining with bioinformatics analysis, literature reports, and enlarged sample verification, we have screened some potential key pairs of lncRNA and target mRNA, and hypothesized that these potential key lncRNAs may interact with their corresponding coding genes to regulate central inflammation and apoptosis. However, further studies should be performed to verify the association between these lncRNAs and target mRNAs, and whether these lncRNA-mRNA axes play an important role in the development of POCD.

## Ethics Statement

All animal experiments were reviewed and approved by West China Hospital Institutional Animal Care and Use Committee.

## Author Contributions

CC, MiL, TZ, JL, and WZ designed the experiments. MaL, RG, QW, ZZ, and HC performed the experiments. MiL, CC, XM, AB, LG, HY, WZ, and TZ analyzed the data. CC, MiL, TZ, JL, and WZ wrote the original manuscript. All authors reviewed the manuscript.

## Conflict of Interest Statement

The authors declare that the research was conducted in the absence of any commercial or financial relationships that could be construed as a potential conflict of interest.

## Footnotes

1 http://www.geneontology.org

2 http://www.genome.jp/kegg/
