# Supplementary material for: Identification of the Potential Key Long Non-coding RNAs in Aged Mice With Postoperative Cognitive Dysfunction
Source: Front Aging Neurosci. 2019 Jul 17;11:181. doi: 10.3389/fnagi.2019.00181 (PMC6650538; doi:10.3389/fnagi.2019.00181)
Supplement: Supplementary file 1 [file Table_1.DOCX]

**TABLE S1 |** The primers used for qRT-PCR analysis in the study.

|  | Forward(5'-3') | Reverse(5'-3') |
| --- | --- | --- |
| ENSMUST00000126434 | GTAAGGATCTAGTATGGCAGGCAG | GCAAGTGAAGCCTTGTTACTTG |
| AK042300.1 | ACAAGGAAGGATGTGACTTGAAAG | TTTAGAGTCCTGCCCAAACCTA |
| AK016122 | GTGCTGTCAGAGAAGACCAGA | GAGCCTTCTCTAAACGCTCCA |
| uc011ysu.1 | TGTCACGGTCAGCTCTGTTC | AGTGCCAATCCCATTGAGGG |
| ENSMUST00000174338 | CATTACAGGCAGGGAAGGACT | CTCTTTACTTCTGAGCCACCTCT |
| Lcn2 | GGAACGTTTCACCCGCTTTG | TTCAGCAGAAAGGGGACGCC |
| Lrg1 | CCTCGACCTTGGGTACAACC | TTGTTTCGGTTGGCGACCAG |
| Rtf1 | TGAGTGGACATTTGGGAGCAA | GGATGAGCGGTCCGATTTCT |
| Rhbg | AGCACCCTCAGTACTTTCGC | TGGACCCAAAGAGGGTTTGT |
| Smad7 | CAAACCAACTGCAGGCTGTC | TGAACTCGTGGTCATTGGGC |
| TNF-α | CTGTGAAGGGAATGGGTGTT | CAGGGAAGAATCTGGAAAGGTC |
| IL-1β | TGCCACCTTTTGACAGTGATG | CATCTCGGAGCCTGTAGTGC |
| CXCL2 | GCCCAGACAGAAGTCATAGC | AGCGAGGCACATCAGGTA |
| CCL2 | CCCCAAGAAGGAATGGGTCC | GTGCTGAAGACCTTAGGGCA |
| CXCL1 | GCACCCAAACCGAAGTCA | AAGCCAGCGTTCACCAGA |
| 18S | TTGACTCAACACGGGAAACC | AGACAAATCGCTCCACCAAC |
