# Supplementary material for: Identification of the Potential Key Long Non-coding RNAs in Aged Mice With Postoperative Cognitive Dysfunction
Source: Front Aging Neurosci. 2019 Jul 17;11:181. doi: 10.3389/fnagi.2019.00181 (PMC6650538; doi:10.3389/fnagi.2019.00181)
Supplement: Supplementary file 5 [file Data_Sheet_1.docx]

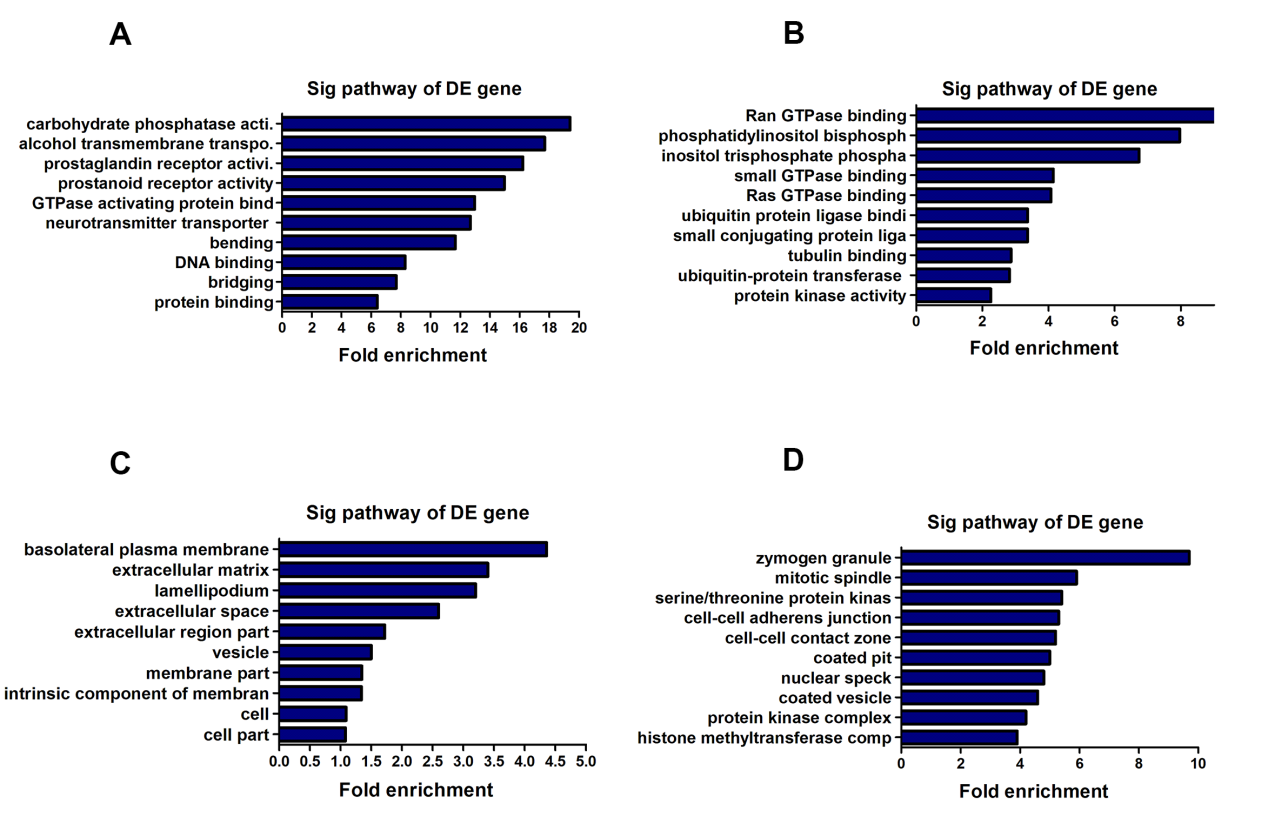


**Figure S1.** The top 10 functional GO terms for molecular function analysis and cellular component analysis. **(A)** The top 10 upregulated functional GO terms for molecular function analysis. **(B)** The top 10 downregulated functional GO terms for molecular function analysis. **(C)** The top 10 upregulated functional GO terms for cellular component analysis. **(D)** The top 10 downregulated functional GO terms for cellular component analysis.
